# Positive Correlation between BMI and Left Ventricle and Atrium Inside Diameter Size in Chinese Type 2 Diabetes Patients with Left Ventricular and Atrial Enlargement

**DOI:** 10.31083/j.rcm2506207

**Published:** 2024-06-04

**Authors:** Jie Zhang, Zhenhua Yang, Xiaoxiang Fan, Qiuping Fei, Yingfei Xi

**Affiliations:** ^1^Department of Endocrinology, Ningbo No.2 Hospital, 315000 Ningbo, Zhejiang, China

**Keywords:** left ventricular enlargement, left atrial enlargement, type 2 diabetes, heart failure, body mass index

## Abstract

**Background:**

Patients with type 2 diabetes mellitus (T2DM) commonly exhibit overlooked left ventricular and atrial hypertrophy. This research identifies potential risk factors and intervention targets.

**Methods:**

T2DM patients with normal ejection fraction values were enrolled, while we eliminated influences on heart size, such as hypertension and coronary heart disease. Variables for each participant, including height, weight, age, body mass index (BMI), and blood biochemistry, were recorded before patients were categorized into four groups based on heart size. Multiple linear regression and Pearson’s correlation analyses were applied to investigate the possible correlations.

**Results:**

Three years of clinical data were collected for each T2DM patient, while patients with incomplete data or interference factors affecting heart size were excluded. BMI, adjusted fasting blood glucose (FBG), glomerular filtration rate (eGFR), and age all showed a significant positive correlation with the inner diameter of the left ventricle and atrium in groups exhibiting hypertrophy.

**Conclusions:**

In T2DM patients, BMI correlated positively with left ventricular enlargement, suggesting its potential role as a risk factor. Weight control may be an effective intervention for left ventricular enlargement, to reduce the likelihood of heart failure.

## 1. Introduction

Type 2 diabetes mellitus (T2DM) represents a worldwide pandemic, with interrelated genetic, metabolic, lifestyle, and environmental factors all contributing to its development [1]. As a common clinical chronic disease, T2DM occurs with metabolic and endocrine disorders [2]. Arteriosclerotic cardiovascular disease (ASCVD) represents the major co-morbidity associated with T2DM and the principal reason for death and disability among patients, thereby creating a huge economic burden [3, 4]. Emerging evidence has indicated that the occurrence of heart failure (HF), the initial manifestation of ASCVD, in patients with diabetes is relatively high [5]. The likelihood of HF in patients with T2DM is 12–57% [6]. Thus, patients with HF are prone to receive worse prognoses than those patients without HF [7]. Type 2 diabetes also represents an independent risk factor of HF since a higher risk of HF is observed in patients suffering from type 2 diabetes [8]. Moreover, the mortality rate of T2DM patients with HF is significantly higher, approximately 11 to 13 times, than those without HF [6]. Obviously, the interaction between type 2 diabetes and HF exists in both occurrence and clinical outcomes. However, the identification of predictors and intervention targets is still required.

Generally, left ventricular hypertrophy (LVH) is the aberrant enlargement of the left ventricular mass due to an increase in cardiomyocyte size, which is modulated by volume and/or pressure overload [9]. In the beginning, physiological LVH is benign and protective, and it regresses when physical activity is reduced or stopped. While pathological LVH is compensative and maladaptive, even evolving toward left ventricular dysfunction and HF [10]. LVH has been indicated as an independent risk factor of HF and it is relatively common in Chinese T2DM patients [11]. In prediabetes, an increase in LV quality can be observed upon impaired glucose tolerance [12]. The left ventricular mass/volume ratio and relative wall thickness are increased in diabetes patients [13]. Thus, it is a representative manifestation of diabetic cardiomyopathy.

Researchers have found that the body mass index (BMI), a well-known measure in daily life, has strong associations with LVH [14]. Vitamin D is a considerable hormone with corresponding receptors in cardiac myocytes, while its deficiency is related to cardiovascular diseases, including LVH. Moreover, parathyroid hormone (PTH) is a predicting variable of LVH, a decrease in which can cause a reduction in left ventricular mass [15]. A positive correlation exists between the left ventricular mass index and fasting blood glucose (FBG) or glycosylated hemoglobin A1c (HbA1c) [16]. In type 2 diabetes and HF patients, high HbA1c is related to an increase in cardiovascular mortality [12]. Insulin sensitivity, C-peptide, homeostasis model assessment index (HOMA-IR), HOMA-β, and serum creatinine (Cr) are also commonly tested variables in studies on LVH [17, 18, 19]. Thus, in this research, these possible variables were examined to explore potential intervention targets.

## 2. Methods

### 2.1 Study Design

A three-year retrospective study was performed in the Ningbo No.2 hospital to evaluate the influence of BMI on LVH in diabetic participants with normal blood pressure (BP) and LVH. Subjects were recruited from January 1st, 2017, to January 1st, 2020. Since it was a retrospective study, we received ethnic approval in 2020 (Ethics No.: YJ-KYSB-NBEY-2019-158-01). The study was conducted according to the guidelines presented in the Declaration of Helsinki.

### 2.2 Study Population

Patients with T2DM were recruited over a 3-year period from the in-patient Department of Endocrinology and Metabolism, Ningbo Hwa Mei Hospital University of Chinese Academy of Sciences (Ningbo No.2 hospital). Patients were recruited based on the diagnostic criteria outlined in 1999 by the World Health Organization (WHO): (1) diabetes symptoms (hyperglycemia, polydipsia, polyuria, weight loss, skin pruritus, decreased visual acuity, etc.) and random blood glucose levels higher than or equal to 11.1 mmol/L; (2) fasting blood glucose levels higher than or equal to 7.0 mmol/L; (3) postprandial blood glucose levels higher than or equal to 11.1 mmol/L after glucose loading.

### 2.3 Inclusion Criteria

(1) Diagnosed with type 2 diabetes in accordance with the current WHO 1999 guidelines, as described above.

(2) Aged 40–70 years.

(3) Blood pressure (BP) <145/90 mmHg at screening visit. However, if BP is out of the range, patients will undergo 24 h recording in order to adequately control the BP.

(4) Echocardiographic LVH: an LV mass index >115 g/$m^{2}$ for men and >95 g/$m^{2}$ for women indexed to body surface area or >48/44 g/m2.7 indexed to height2.7.

(5) N-terminal B-type natriuretic peptide (NT-BNP) and fasting glucose tests were performed.

### 2.4 Exclusion Criteria

(1) Acute infection or history of immunodeficiency virus.

(2) Diagnosed with clinical heart failure, tumor, diabetic ketoacidosis, or type 1 diabetes mellitus.

(3) Serum sodium or potassium beyond the normal range.

(4) Pregnant or breastfeeding patients.

(5) Left ventricular systolic dysfunction—left ventricular ejection fractions (LVEF) <45% (last outcome within six months).

(6) Estimated glomerular filtration rate (eGFR) <60 mL/min/1.73 $m^{2}$ (last outcome within six months).

(7) Body weight >150 kg (unsuitable for a magnetic resonance imaging (MRI) scanner).

(8) Liver function tests >3 times the upper limit of normal (last tests and documented laboratory examination within six months).

### 2.5 Echocardiography

Echocardiography was screened by a Philips Epiq 7 machine (Andover, Massachusetts, USA) following the standard American Society of Echocardiology (ASE) criteria. The left atrium (LA) wall thickness, interventricular septum (IVS), left ventricular (LV), and right ventricular (RV) were determined upon M-mode images. Furthermore, the left ventricular ejection fraction (EF) and fraction shortening (FS) were calculated.

### 2.6 Anthropometric and Biochemical Analyses

Age, weight, height, and BMI values were recorded for each participant. After a 5-minute rest, systolic blood pressure (SBP) and diastolic blood pressure (DBP) were examined, while a second measurement was conducted after a 10-minute interval. Fasting blood samples were drawn between 08:00 and 09:00 for biochemical analysis. FBG (blood glucose kits, Zhejiang PORABIO, Hangzhou, China) was assessed by the SIEMENS ADVIA2400 access immunoassay system via the oxygen electrode method. Cr and blood–urea–nitrogen (BUN) were analyzed by the SIEMENS ADVIA2400 analyzer (Abbott, Wiesbaden, Germany) using Zhejiang PORABIO reagents. NT-BNP was examined by enhanced immunochemical luminescence via a SIEMENS ADVIA2400 immunoanalyzer. eGFR was quantified using the Modification of Diet in Renal Disease (MDRD) GFR equation [20].

### 2.7 Statistical Analysis

Data analysis was conducted using SPSS19.0 software (IBM Corp., Armonk, NY, USA) with the continuous variables expressed as mean ± standard deviation (SD), similar to in previous studies [21]. Experiments were performed in triplicate. The least significant difference tests (LSD-t) were applied to analyze variables in groups. Further, the association between two variables was explored by multiple linear regression analysis. Data with *p* values < 0.05 were considered statistically significant.

## 3. Results 

### Basic Characteristics of Participants and the Association of BMI with Risk

**Fig. 1.**
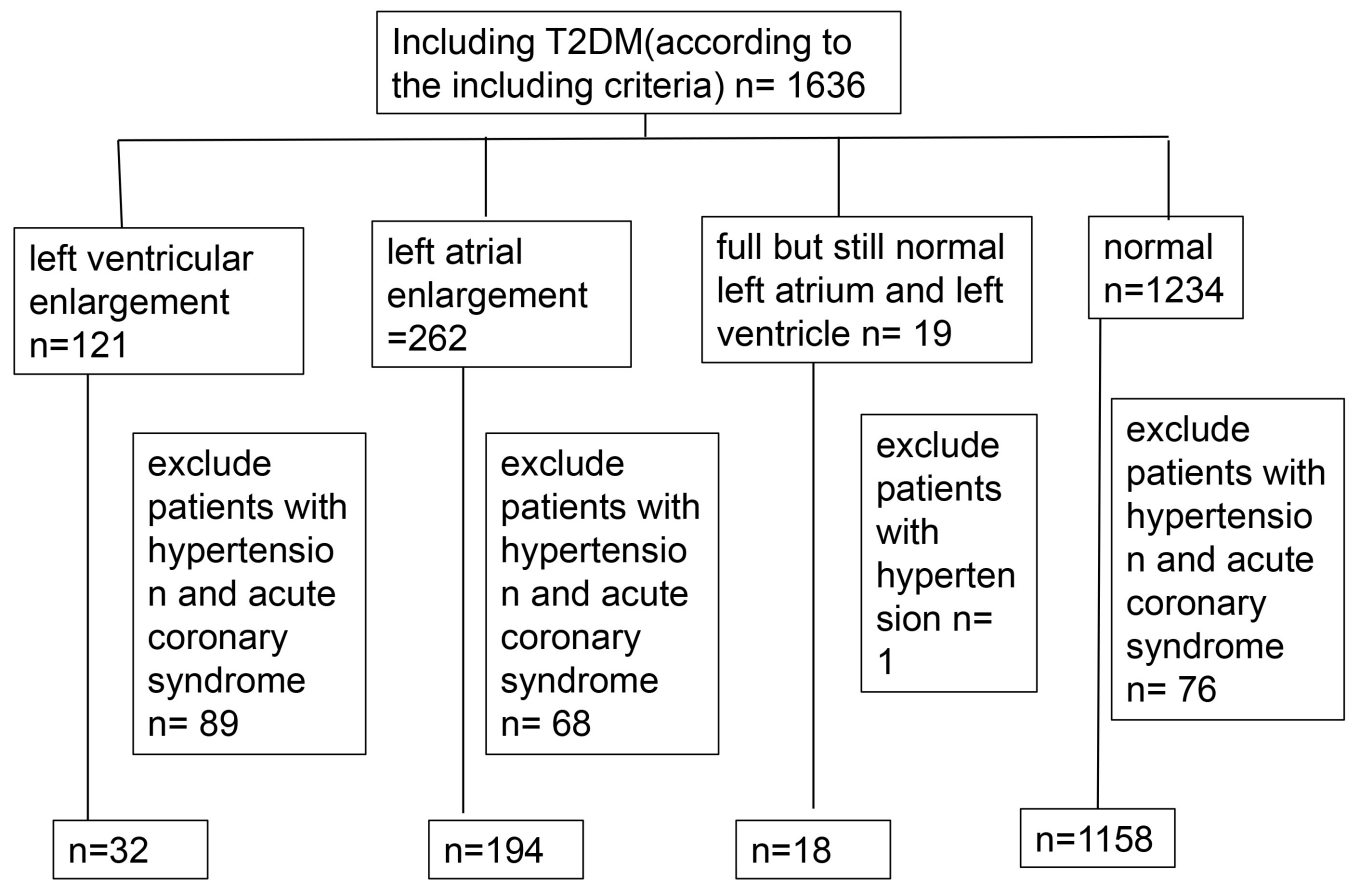
**Flowchart of included and excluded subjects.** T2DM, type 2 diabetes.

**Fig. 2.**
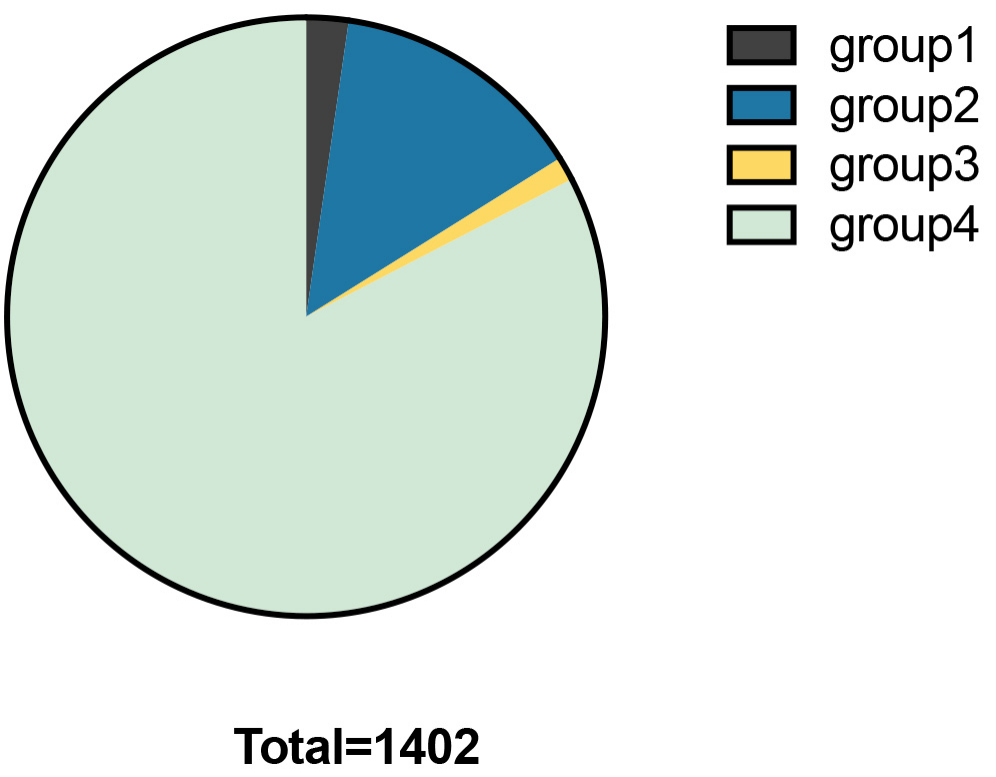
**Percentages in each group**.

The clinical data of 1402 patients with T2DM were included, while those with incomplete data were excluded (Figs. 1,2). After influencing factors on heart size, such as hypertension, cardiomyopathy, dilated cardiomyopathy, and coronary heart disease, were eliminated, patients were mainly divided into four groups: Group 1: left ventricular enlargement (left ventricular diameter larger than 56 mm), included 32 subjects (2.28%); Group 2: left atrial enlargement (left atrium diameter larger than 31 mm), included 194 subjects (13.84%); Group 3: full but still normal left atrium and left ventricle, included 18 subjects (left atrium diameter is between 30 and 31 mm and left ventricular diameter in systolic stage is between 25 and 26 mm, left ventricular diameter of female patients in diastolic stage is between 50 and 51 mm, left ventricular diameter of female patients in diastolic stage is between 55 and 56 mm), (1.28%); Group 4: normal heart size (left atrium diameter is between 23 and 38 mm and left ventricular diameter in systolic stage is between 13 and 25 mm, left ventricular diameter of female patients in diastolic stage is between 35 and 50 mm, left ventricular diameter of female patients in diastolic stage is between 45 and 55 mm), included 1158 subjects (82.60%).

As shown in Table 1, the Group 1 characteristics, including FBG, weight, height, BMI, eGFR, Cr, BUN, NT-BNP, LA, IVS, LV, RV, EF, and FS were measured.

**Table 1.** **Characteristics of Group 1**.

Characteristics		n
FBG (mmol/L)	9.13 ± 5.19	32
Weight (kg)	63.13 ~ 77.97	32
Height (m)	1.67 ± 0.82	32
BMI (kg/$m^{2}$)	25.61 ± 2.97	32
Age (year)	57.91 ± 7.52	32
eGFR (mL/min)	104.68 ± 55.62	32
Cr (μmol/L)	81.64 ± 26.67	32
BUN (mmol/L)	6.28 ± 4.56	32
NT-BNP (ng/L)	182.5 ± 7904.75	32
LA (mm)	43.41 ± 5.28	32
IVS (mm)	9.73 ± 2.06	32
LV (mm)	56.66 ± 8.35	32
RV (mm)	21.81 ± 3.29	32
EF (%)	56.06 ± 12.16	32
FS (%)	30.38 ± 8.14	32

FBG, fasting blood glucose; BMI, body mass index; eGFR, estimated glomerular filtration rate; Cr, creatinine; BUN, blood–urea–nitrogen; NT-BNP, N-terminal B-type natriuretic peptide; LA, left atrium; IVS, interventricular septum; LV, left ventricular; RV, right ventricular; EF, ejection fraction; FS, fractional shortening.

Moreover, 194 subjects were enrolled in Group 2. As shown in Table 2, the basic characteristics, such as FBG, BMI, age, eGFR, NT-BNP, LA, IVS, LV, RV, EF, and FS were again assessed.

**Table 2.** **Characteristics of Group 2**.

Characteristics		n
FBG (mmol/L)	[5, 11.62]	194
BMI (kg/$m^{2}$)	26.30 ± 3.47	194
Age (year)	[54.75, 67]	194
eGFR (mL/min)	[88.9, 137.6]	194
NT-BNP (ng/L)	[156, 823]	194
LA (mm)	[41, 45]	194
IVS (mm)	[9, 11]	194
LV (mm)	[46, 51]	194
RV (mm)	[21, 23]	194
EF (%)	[62, 69]	194
FS (%)	36.18 ± 4.02	194

FBG, fasting blood glucose; BMI, body mass index; eGFR, estimated glomerular filtration rate; NT-BNP, N-terminal B-type natriuretic peptide; LA, left atrium; IVS, interventricular septum; LV, left ventricular; RV, right ventricular; EF, ejection fraction; FS, fractional shortening.

As presented in Table 3, weight, height, BMI, age, NT-BNP, LA, IVS, LV, RV, EF, and FS were also estimated for Group 3. Finally, the same characteristics were also recorded for the 1158 participants in Group 4; presented in Table 4.

**Table 3.** **Characteristics of Group 3**.

Characteristics		n
Weight (kg)	68.94 ± 12.85	18
Height (m)	166.06 ± 8.94	18
BMI (kg/$m^{2}$)	24.83 ± 3.08	18
Age (year)	58.50 ± 7.28	18
NT-BNP (ng/L)	77.75± 416.75	18
LA (mm)	38.17± 4.12	18
IVS (mm)	9.72± 0.89	18
LV (mm)	47.61± 4.57	18
RV (mm)	21.67 ± 2.87	18
EF (%)	66.33 ± 4.69	18
FS (%)	35.61 ± 3.62	18

BMI, body mass index; NT-BNP, N-terminal B-type natriuretic peptide; LA, left atrium; IVS, interventricular septum; LV, left ventricular; RV, right ventricular; EF, ejection fraction; FS, fractional shortening.

**Table 4.** **Characteristics of Group 4**.

Characteristics		n
Weight (kg)	56 ~ 71	1158
Height (m)	159 ~ 170	1158
BMI (kg/$m^{2}$)	21.25 ~ 25.86	1158
Age (year)	52 ~ 64	1158
LA (mm)	32 ~ 37	1158
IVS (mm)	8 ~ 10	1158
LV (mm)	44 ~ 50	1158
RV (mm)	19 ~ 22	1158
EF (%)	63 ~ 70	1158
FS (%)	34 ~ 39	1158

BMI, body mass index; LA, left atrium; IVS, interventricular septum; LV, left ventricular; RV, right ventricular; EF, ejection fraction; FS, fractional shortening.

Relationships between LV and FBG, BMI, Age, eGFR, and weight in Group 1.

**Fig. 3.**
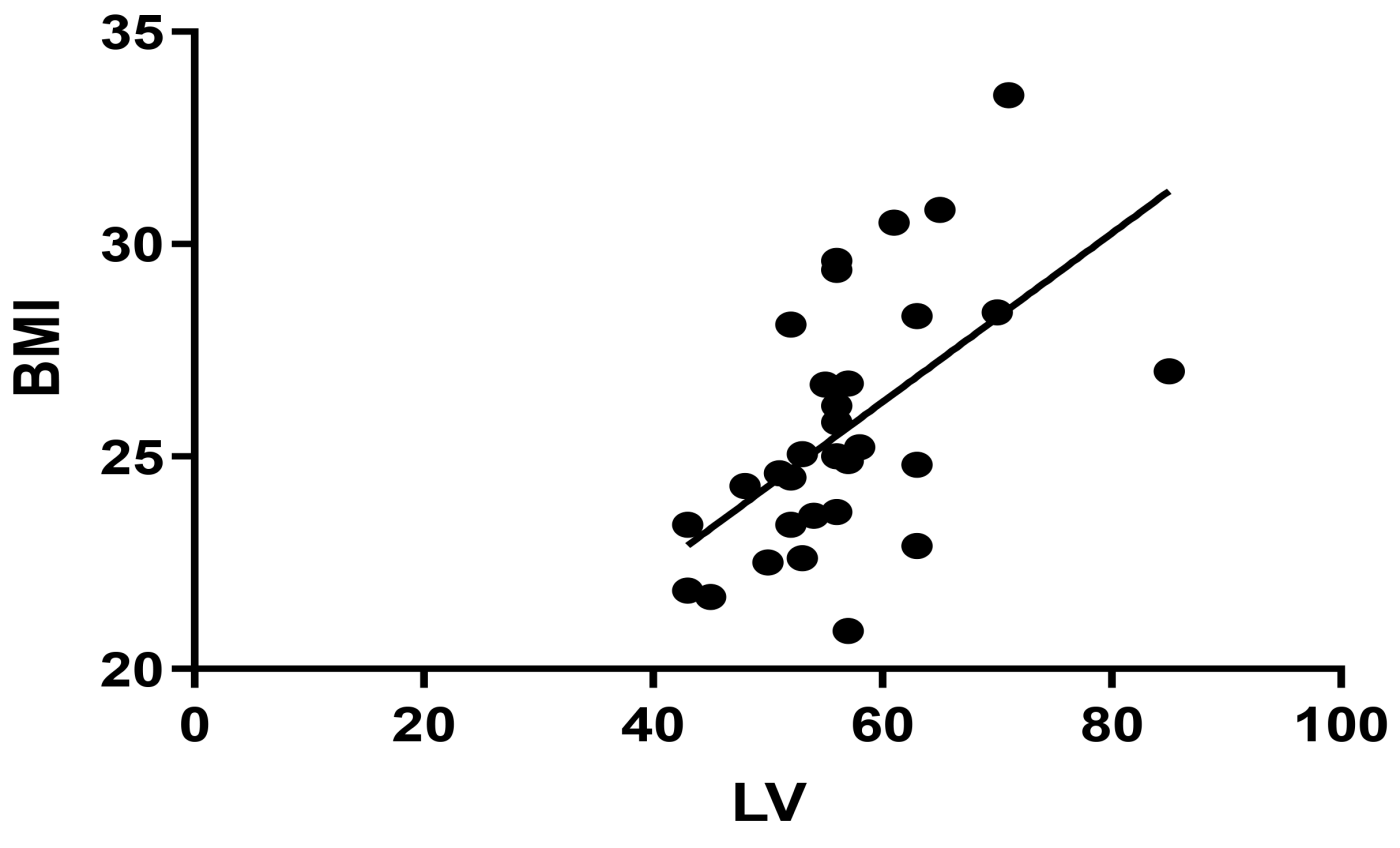
**Pearson correlation between BMI and LV in Group 1 (r = 0.558, *p* = 0.001). **BMI, body mass index; LV, left ventricular.

In the multiple linear regression analysis (shown in Table 5), BMI was a predictor of LV (β = 0.614, *p* = 0.001) when used as the dependent variable, although not with FBG, eGFR, age, and weight as independent variables. The Pearson correlation coefficient showed a positive correlation between BMI and LV (r = 0.558, *p* = 0.001) (shown in Fig. 3). The multiple linear regression analysis for LA and FBG, BMI, Age, and eGFR in Group 2.

**Table 5.** **The multiple linear regression between LV and BMI, FBG, eGFR, and age in Group 1 (β = 0.614, *p* = 0.001)**.

	β	*p* value
FBG	–0.257	0.189
BMI	0.614	0.001
Age	–0.137	0.423
eGFR	–0.174	0.268

LV, left ventricular; FBG, fasting blood glucose; BMI, body mass index; eGFR, estimated glomerular filtration rate.

**Fig. 4.**
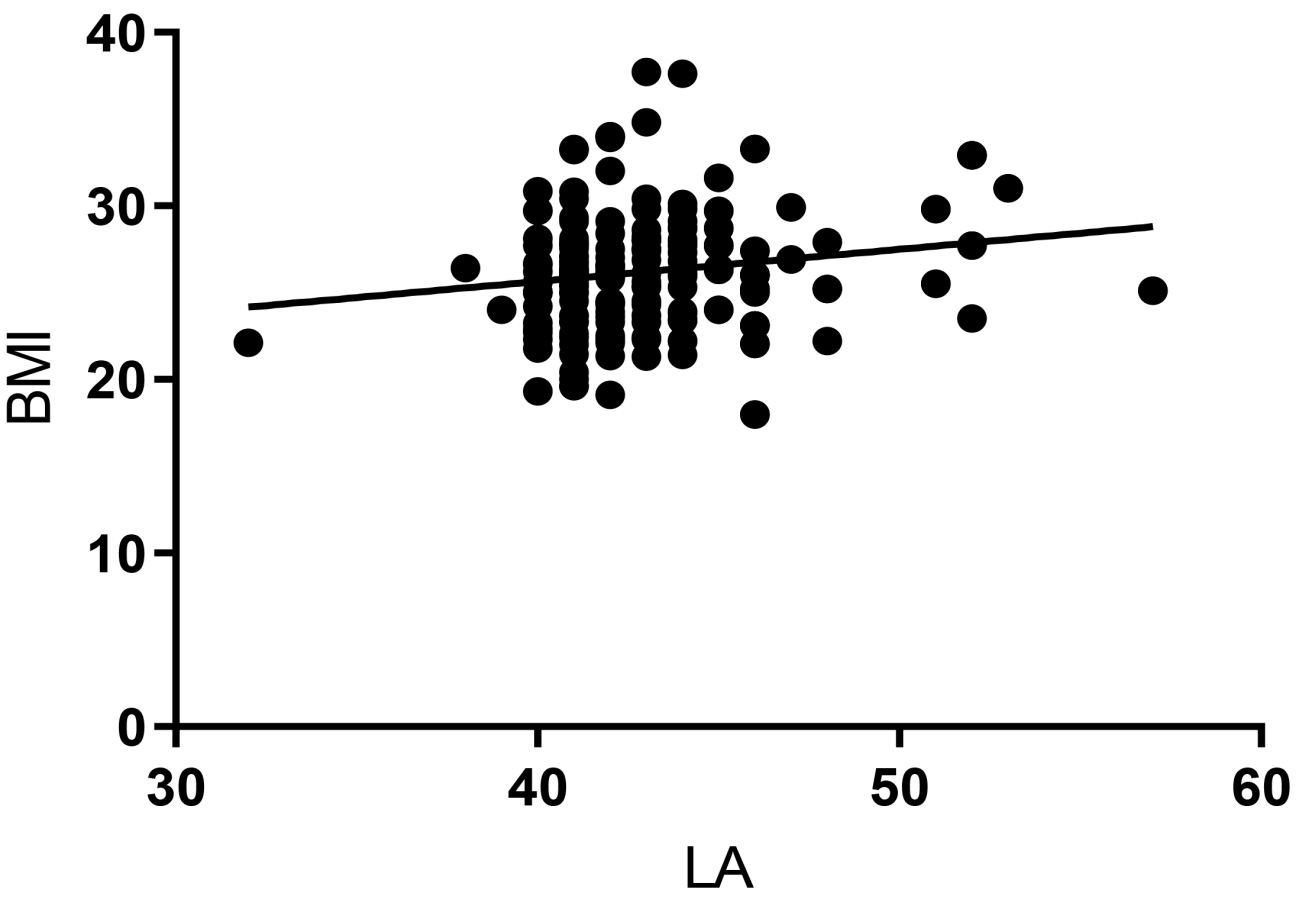
**Pearson correlation between BMI and LA in Group 2 (r = 0.182, *p* = 0.011).** BMI, body mass index; LA, left atrium.

Similarly, BMI was a predictor of LA (β = –0.185, *p* = 0.011) in the multiple linear regression analysis (shown in Table 6), as the dependent variable, while not with FBG, eGFR, age, and weight as independent variables. The Pearson correlation coefficient showed a positive correlation between BMI and LA (r = 0.182, *p* = 0.011) (shown in Fig. 4).

**Table 6.** **The multiple linear regression between LA and BMI, FBG, eGFR, age, and weight in Group 2 (β = 0.185, *p* = 0.011)**.

	β	*p* value
FBG	0.019	0.798
BMI	0.185	0.011
Age	–0.003	0.963
eGFR	–0.035	0.631

BMI, body mass index; LA, left atrium; FBG, fasting blood glucose; eGFR, estimated glomerular filtration rate.

## 4. Limitations

This is a retrospective research study, meaning a causality relationship could not be provided between LV and BMI or between LA and BMI.

## 5. Discussion

This study found that 10% of patients with normal ejection fraction values but no clinical symptoms of heart failure had left ventricular volume dilation. Following, the patients with hypertension, coronary heart disease, acute myocardial infarction, and cardiomyopathy were removed, and the influence factors were adjusted. Among the potential influence factors, such as FBG, age, eGFR, and BMI, only BMI was positively related to left ventricular volume in patients with either left ventricular or left atrial enlargement. This is in accordance with the finding that the introduction of novel therapies, such as sodium-glucose co-transporter 2 (SGLT2i) improved the risk of HF in diabetic patients [21]. Moreover, SGLT2i has also been proven to ameliorate cardiac remodeling, previously characterized by the improvement of left ventricular global longitudinal strain (LV-GLS) and myocardial work efficiency in hearts with preserved ejection fraction, while BMI owns a positive correlation with left ventricular size [22], which implies that patients with higher BMIs possess bigger left ventricular volumes and HF risks [23]. Thus, BMI may be an early predictor of HF in type 2 diabetes patients. Moreover, controlling BMI to prevent obesity may be a possible method to prevent left ventricular enlargement and HF in diabetic patients. However, since this study was a retrospective study and data relating to glycosylated hemoglobin was incomplete, performing linear regression analysis was not suitable for analyzing causality. Therefore, further prospective controlled studies are needed to improve and support the above phenomenon.

Previous papers have studied the effect of body weight in diabetic rats. Body weight gain and feed intake were shown to be decreased by fructans in diabetic rats [24]. Insulin can also decrease body weight and food intake in diabetic rats [25]. The body weight of diabetic rats treated by gastrectomy is significantly lower than in sham-operated diabetic rats [26]. Body weight is a common symptom in diabetic rats, which is generally recognized as a testing index. We also observed that increases in weight in diabetic rats caused sharp increases in heart weight, the transverse and longitudinal diameters of the heart, the number of fibroblasts, and the expression of collagen IV in cardiomyocytes. Consistent with the results of population studies, increases in body weight accelerated pathological changes in the myocardium and aggravated LVH in diabetic rats.

It has already been reported that inflammatory reactions are a mechanism that leads to LVH [27]. The accumulation of globotriaosylceramide induces LVH by releasing pro-inflammatory cytokines and growth-promoting factors as well as inducing oxidative stress [28]. In renovascular hypertensive rats, treatment of olmesartan mitigates LVH and reduces IL-6 levels [28]. One research study investigated the function of inflammation in epicardial adipose tissue in heart diseases [29]. Accumulating data suggest that type 2 diabetic patients present with hyperglycemia and metabolic disorders, such as lipid metabolism and protein metabolism disorders [30, 31]. It is not a simple disease but a series of syndromes of multiple organs caused by metabolic disorders [32]. Insulin resistance exerts a vital role in the development of type 2 diabetes, which is closely related to BMI augmentation [33]. For patients with type 2 diabetes, BMI is of great significance to left ventricular volume dilation and increases in HF risk, not only in the heart but also in highlighting the importance of weight management in metabolic balance. Although some research showed that obesity may have an influence on vascular aging and arterial stiffness [34, 35], the subjects we included had mostly normal BMI or were overweight, and only in a few cases, were they diagnosed as obese.

It is indicated from a study that in patients suffering from type 2 diabetes, BMI is an independent risk for LVH [36]. Additionally, a positive correlation of body weight with heart size in type 2 diabetic rats was observed [37]. These data hint that body weight is a possible independent risk for LVH in type 2 diabetic patients. In the subsequent exploration of the in-depth mechanisms involved, we detected that the follow-up weight and the content of myocardial inflammatory factors in rats were markedly increased, and that pyroptosis existed in pericardial adipocytes. It has been proposed that the inflammatory response is a pathogenesis that accounts for the complications associated with type 2 diabetes [38].

## 6. Conclusions

As far as the literature we retrieved, no previous research has been conducted on the etiology and mechanism of left ventricular volume dilation and HF in patients with type 2 diabetes. This work provides new ideas for the early prevention of left ventricular volume dilation, HF, and ASCVD in type 2 diabetic patients.

## Data Availability

The datasets used and/or analyzed during the current study are available from the corresponding author on reasonable request.
